# Synthesis and evaluation of *N*-isopropyl-*p*-[^11^C]methylamphetamine as a novel cerebral blood flow tracer for positron emission tomography

**DOI:** 10.1186/s13550-020-00702-5

**Published:** 2020-10-01

**Authors:** Jun Toyohara, Norihiro Harada, Takeharu Kakiuchi, Hiroyuki Ohba, Masakatsu Kanazawa, Tetsuro Tago, Muneyuki Sakata, Kiichi Ishiwata

**Affiliations:** 1 Research Team for Neuroimaging, Tokyo Metropolitan Institute of Gerontology, 35-2 Sakae-cho, Itabashi-ku, Tokyo, 173-0015 Japan; 2 Central Research Laboratory, Hamamatsu Photonics, 5000 Hiraguchi, Hamakita-ku, Hamamatsu, 434-8601 Japan; 3 Southern TOHOKU Drug Discovery and Cyclotron Research Center, Southern TOHOKU Research Institute for Neuroscience, 7-61-2 Yatsuyamada, Koriyama, 963-8052 Japan; 4 Department of Biofunctional Imaging, Fukushima Medical University, 1 Hikariga-oka, Fukushima, 960-1295 Japan

**Keywords:** Phenylalkylamine, Cerebral blood flow, Positron emission tomography, Non-human primate, Carbon-11

## Abstract

**Introduction:**

Increases in fasting plasma glucose (PG) levels lead to a decrease in 2-deoxy-2-[^18^F]fluoro-d-glucose ([^18^F]FDG) uptake in the normal brain, especially in the precuneus, resulting in an Alzheimer’s disease (AD)-like uptake pattern. Therefore, patients with higher PG levels, such as those with diabetes, can be erroneously diagnosed with AD when positron emission tomography (PET) imaging is done using [^18^F]FDG, due to reduced uptake of [^18^F]FDG in the precuneus. To help avoid an erroneous diagnosis of AD due to differences in glucose metabolism, evaluating cerebral blood flow (CBF) in the brain is useful. However, current techniques such as single photon emission computed tomography (SPECT) and [^15^O]H_2_O PET have limitations regarding early diagnosis of AD because the images they produce are of low resolution. Here, we developed a novel CBF PET tracer that may be more useful than [^18^F]FDG for diagnosis of AD.

**Methods:**

We synthesized and evaluated *N*-isopropyl-*p*-[^11^C]methylamphetamine ([^11^C]**4**) as a carbon-11-labeled analogue of the standard CBF SPECT tracer *N*-isopropyl-*p*-[^123^I]iodoamphetamine. Fundamental biological evaluations such as biodistribution, peripheral metabolism in mice, and brain kinetics of [^11^C]**4** in non-human primates with PET with successive measurement of [^15^O]H_2_O were performed.

**Results:**

[^11^C]**4** was synthesized by methylation of the corresponding tributyltin precursor (**2**) with [^11^C]MeI in a palladium-promoted Stille cross-coupling reaction. The brain uptake of [^11^C]**4** in mice peaked at 5–15 min after injection and then promptly decreased. Most radioactivity in the brain was detected in the unchanged form, although in the periphery, [^11^C]**4** was rapidly metabolized to hydrophilic components. Acetazolamide (AZM) treatment significantly increased the brain uptake of [^11^C]**4** without affecting the blood levels of radioactivity in mice. Preliminary kinetics analysis showed that the *K*_1_ of [^11^C]**4** reflected regional CBF in a vehicle-treated monkey, but that the *K*_1_ did not reflect CBF in higher flow regions after AZM loading.

**Conclusion:**

[^11^C]**4** is a potential novel CBF PET tracer. Further validation studies are needed before [^11^C]**4** can be used in humans.

## Introduction

Cerebral glucose metabolism primarily reflects synaptic transmission. Therefore, fluorine-18-labeled 2-deoxy-2-[^18^F]fluoro-D-glucose ([^18^F]FDG) has been used in positron emission tomography (PET) as a tool for studying neuronal function in health and diseases [1–5]. [^18^F]FDG-PET is a well-established tool for diagnosis of Alzheimer’s disease (AD) and mild cognitive impairment, which may progress to AD, because glucose hypometabolism reflects a loss of synaptic function and/or density. In patients with AD or mild cognitive impairment, a prominent decrease in [^18^F]FDG uptake can be observed in the posterior cingulate, precuneus, and/or temporoparietal cortices, a pattern called an AD pattern [6]. Some lines of evidence suggest that increased plasma glucose (PG) levels can reduce the cerebral uptake of [^18^F]FDG, resulting in an AD-like cerebral distribution pattern of [^18^F]FDG in cognitively normal subjects [7, 8], cognitively normal diabetes mellitus (DM) patients [9], and carbon-11-labeled Pittsburgh Compound B-negative mild cognitive impairment (non-AD) patients [10]. This AD-like pattern can appear even in an individual with slightly higher levels of fasting PG (100–110 mg/dL) [11]. Furthermore, this AD-like pattern is reversible [10, 12] and is due more to PG levels than plasma insulin levels [12]. These studies imply that an individual with higher PG levels can be erroneously diagnosed with AD when using [^18^F]FDG-PET. Although the detailed mechanisms of the AD-like pattern of decreased [^18^F]FDG uptake during the hyperglycemic state are unknown, theoretically, [^18^F]FDG competes with glucose on glucose transporters and hexokinases [13] and hence results in a net reduction in the glucose metabolic rate in the brain [14].

The prevalence of AD and DM inevitably increases with age [15, 16]. In addition, due to the rapid increase in the aging population, more patients have both DM and dementia. Therefore, an alternative tracer to [^18^F]FDG for diagnosis of dementia in patients with DM is needed. To avoid an erroneous diagnosis of AD due to differences in glucose metabolism, evaluating cerebral blood flow (CBF) in the brain is useful [17–19]. Neuronal activity is tightly coupled to regional CBF changes [20, 21]. However, current techniques such as single photon emission computed tomography (SPECT) and [^15^O]H_2_O/[^15^O]O_2_-gas-PET have limitations for early diagnosis of AD because the images they produce are of low resolution. Therefore, we aimed to develop a novel CBF PET tracer labeled with a conventional short-lived, lower positron range radionuclide, such as ^11^C or ^18^F, that can be used instead of [^18^F]FDG for diagnosis of AD. In this study, we synthesized and evaluated *N*-isopropyl-*p*-[^11^C]methylamphetamine ([^11^C]**4**) as a carbon-11-labeled analogue of the standard CBF SPECT tracer *N*-isopropyl-*p*-[^123^I]iodoamphetamine ([^123^I]IMP). Fundamental biological evaluations such as biodistribution, peripheral metabolism in mice, and successive [^15^O]H_2_O-PET imaging in non-human primates with [^11^C]**4** were also performed.

## Materials and methods

### General

1-(4-Methylphenyl)-propan-2-one (**3**) was purchased from Alfa Aesar (Lancashire, UK). *N*-Isopropyl-*p*-iodoamphetamine (IMP, **1**) was provided by Nihon Medi-Physics (Tokyo, Japan). Acetazolamide (AZM) was purchased from Sigma-Aldrich (St. Louis, MO). ^1^H nuclear magnetic resonance (NMR) spectra were recorded in CDCl_3_ as a solvent using tetramethylsilane as an internal standard on AV400M spectrometers (Bruker, Billerica, MA). Multiplicities are indicated as s (singlet), d (doublet), t (triplet), sext (sextet), or m (multiplet). High-resolution mass spectrometry (HRMS) spectra were recorded on a micrOTOF-Q (Bruker). All isolated materials were shown to be pure with NMR (free of obvious impurities) and thin-layer chromatography (TLC; homogeneous material). All other reagents and solvents were purchased commercially and used as received. Determination of Pd and Cu residue in the final product was performed at Shimadzu Techno-Research (Kyoto, Japan).

Eight-week-old male ddY mice were purchased from Japan SLC (Hamamatsu, Japan). The animals were allowed to acclimate to the laboratory environment for at least 1 week prior to use. The Animal Care and Use Committee of the Tokyo Metropolitan Institute of Gerontology approved the animal studies (Approval No. 18015). The PET study with monkeys was performed at the Central Research Laboratory of Hamamatsu Photonics (Hamamatsu, Japan). Three healthy male monkeys (*Macaca mulatta*; Hamri, Koga, Japan) were used for the study. Monkeys were individually housed in National Institutes of Health (NIH) standard adapted stainless steel cages in a controlled room with a temperature of 24 ± 4 °C, humidity of 50 ± 20%, and a 14-h light/10-h dark cycle. They were fed 120 g of chow (Certified Primate Diet 5048, PMI Nutrition, St. Louis, MO) in the morning and 100 g of raw sweet potato in the evening. Weight (7.5 ± 1.2 kg) and behavior of the animals were monitored during the study. The study was carried out in accordance with the recommendations of the US NIH and the guidelines of the Ethics Committee of the Central Research Laboratory, Hamamatsu Photonics (Approval No. HPK-2019-24) and the Institutional Animal Care and Use Committee of Tokyo Metropolitan Institute of Gerontology (Approval No. 19008).

### Chemistry

**Fig. 1:**
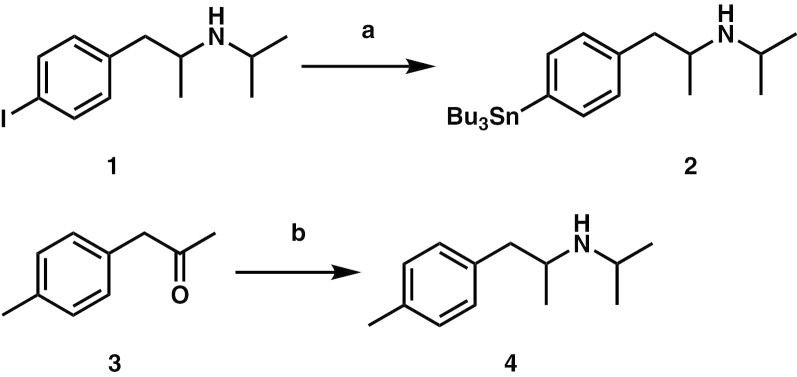
Synthesis scheme of 1-(4-tributylstannyl)phenyl)-*N*-isopropylpropane-2-amine (**2**) and *N*-isopropyl-*p*-methylamphetamine (**4**). Reagents and conditions: (**a**) (Bu_3_Sn)_2_, Pd(PPh_3_)_4_, PhMe, reflux, 16 h. (**b**) Isopropylamine, sodium triacetoxyborohydride, AcOH, room temperature, 3 days

The tributyltin precursor (**2**) was synthesized from **1** with a yield of 40% as shown in Fig. 1. The reference standard (**4**) was prepared from **3** with a yield of 73%. Experimental details and the characterization of compounds are described below.

#### 1-(4-Tributylstannyl)phenyl)-*N*-isopropylpropane-2-amine (2)

To a stirred solution of **1** (3.50 g, 11.5 mmol) in toluene (115 mL), bis(tributyltin) (10.0 g, 17.3 mmol) and tetrakis(triphenylphosphine)palladium(0) (0.67 g, 0.58 mmol) were added at room temperature under an argon atmosphere. The solution was then refluxed for 16 h. The palladium catalyst was filtered off, and toluene was removed in a rotary evaporator. The residue was purified with column chromatography (silica, EtOAc), yielding a clear, colorless solid **2** (2.15 g, 40%). The purity of **2** was assessed with analytic high-performance liquid chromatography (HPLC; retention time (r.t.), 5.4 min; purity, 98.9%). HPLC was performed with an Inertsil SIL 100A column (4.6-mm inner diameter (i.d.) × 150-mm length, 5 µm, GL Sciences, Tokyo, Japan). Elution was conducted using CH_3_Cl/MeOH (98/2, v/v) at a flow rate of 1 mL/min and monitored at 254 nm.

^1^H-NMR (400 MHz, CDCl_3_): *δ* 7.46–7.34 (m, 2H), 7.22–7.18 (m, 2H), 3.76–3.65 (m, 3H), 3.10–3.04 (m, 1H), 1.68 (d, *J* = 6.5 Hz, 3H), 1.59–1.47 (m, 12H), 1.32 (sext, *J* = 7.3 Hz, 6H), 1.12–0.95 (m, 6H), 0.88 (t, *J* = 7.3 Hz, 9H).

HRMS: Calcd. for C_24_H_45_NSn [M + H]^+^ 468.2652; found 468.2630.

#### *N*-Isopropyl-*p*-methylamphetamine (4)

To an ice-cold solution of **3** (2.70 g, 18.2 mmol) in CH_2_Cl_2_ (64 mL), isopropylamine (1.18 g, 20.0 mmol), sodium triacetoxyborohydride (5.40 g, 25.5 mmol), and AcOH (1.09 g, 18.2 mmol) were added and stirred at room temperature for 3 days. After adding water, the resultant aqueous phase was brought to pH 9 by addition of 5 N NaOH and was then extracted with CH_3_Cl. The combined organic layer was dried with MgSO_4_ and concentrated *in vacuo*. The residue was purified with column chromatography (silica, heptane/EtOAc = 50/50), yielding a clear, colorless oil **4** (2.55 g, 73%). The purity of **4** was assessed with analytic HPLC (r.t., 8.2 min; purity, 99.3%). HPLC was performed with an Inertsil ODS-3 column (4.6-mm i.d. × 250-mm length, 5 µm, GL Sciences). Elution was conducted with MeCN/water/trifluoroacetic acid (30/70/0.1, v/v/v) at a flow rate of 1 mL/min and monitored at 254 nm.

^1^H-NMR (400 MHz, CDCl_3_): *δ* 7.10 (d, *J* = 8.1 Hz, 2H), 7.06 (d, *J* = 8.2 Hz, 2H), 2.95 (m, 2H), 2.71 (m, 1H), 2.52 (m, 1H), 2.32 (s, 3H), 1.05 (d, *J* = 6.1 Hz, 3H), 1.01 (d, *J* = 4.1 Hz, 3H), 0.97 (d, *J* = 6.2 Hz, 3H).

HRMS: Calcd. for C_13_H_22_N [M + H]^+^ 192.1747; found 192.1733.

### Radiochemistry

**Fig. 2:**
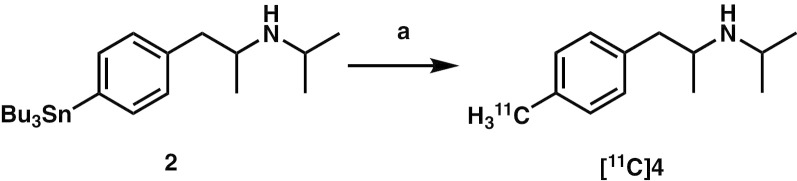
Synthesis of *N*-isopropyl-*p*-[^11^C]methylamphetamine ([^11^C]**4**). Reagents and conditions: (**a**) [^11^C]MeI, Pd_2_(dba)_3_, (*o*-Tol)_3_P, K_2_CO_3_, CuCl, DMF, 100 °C, 5 min

[^11^C]**4** was synthesized by methylation of the tributyltin precursor with [^11^C]MeI in a palladium-promoted Stille cross-coupling reaction as previously described (Fig. 2) [22].

Experimental details and the characterization of compounds are described below. [^11^C]CO_2_ was produced by proton irradiation of nitrogen gas at 50 μA for 30 min using the HM-20 cyclotron (Sumitomo Heavy Industries, Tokyo, Japan). [^11^C]MeI was produced from [^11^C]CO_2_ with an automated system (Sumitomo Heavy Industries). A solution of tri(*o*-tolyl)phosphine (3.7 mg, 12 µmol) in *N*,*N*-dimethylformamide (DMF) (0.15 mL) and the precursor (0.7 mg, 1.5 µmol) in DMF (0.1 mL) was prepared. Immediately before the end of irradiation, this solution was added to a dry septum-equipped vial containing a mixture of CuCl (1.2 mg, 12 µmol), K_2_CO_3_ (1.7 mg, 12 µmol), and tris(dibenzylideneacetone)dipalladium(0) (2.8 mg, 3.0 µmol). The [^11^C]MeI produced was trapped in the reaction mixture of DMF (0.25 mL) with air cooling. The reaction mixture was heated at 100 °C for 5 min. After adding 1.3 mL HPLC eluent [MeCN/50 mM AcOH/50 mM AcONH_4_, (30/35/35, v/v/v)], the reaction mixture was passed through Fine Filter F (Forte Grow Medical, Sano, Japan) equipped with glass fiber wool, followed by injection onto the preparative HPLC: YMC-Pack Pro C18 RS S-5 µm (10-mm i.d. × 250-mm length, YMC, Kyoto, Japan) with a mobile phase of MeCN/50 mM AcOH/50 mM AcONH_4_ (30/35/35, v/v/v) at a flow rate of 5.0 mL/min (ultraviolet (UV) detector at 228 nm). The r.t. of [^11^C]**4** was 6.5 min. The fraction of [^11^C]**4** was collected in a flask containing 0.1 mL of 250 mg/mL ascorbate injection (Nipro Pharma, Osaka, Japan) and evaporated to dryness. The residue was dissolved in physiological saline containing 0.125% (v/v) polyoxyethylene (20) sorbitan monooleate (polysorbate 80) (MP Biomedicals, Santa Ana, CA), and the solution was filtered through a 0.22-µm membrane filter (Millex GV, Merck Millipore, Billerica, MA). Radiochemical purity was analyzed with HPLC using a Titan C18 column (2.1-mm i.d. × 50-mm length, Sigma-Aldrich) with a mobile phase of MeCN/50 mM AcOH/50 mM AcONH_4_ (20/40/40, v/v/v) at a flow rate of 0.25 mL/min (UV detector at 220 nm). The r.t. of [^11^C]**4** was 5.2 min. To determine molar activity, the mass (µmol) of the radioligand with known radioactivity (GBq) was determined for the radioligand with HPLC comparison of UV absorbance at 220 nm with that of known concentrations of the corresponding non-radioactive compound.

The residual amounts of Pd and Cu in the final product were analyzed with an inductivity coupled plasma mass spectrometer (Agilent 7700x, Agilent, Santa Clara, CA). The detection limit is about 100 ng/mL.

### Octanol–water partition coefficient

A mixture of [^11^C]**4** in 4 mL octanol and an equivalent volume of 10 mM phosphate-buffered saline (pH 7.4) were vortexed three times for 20 s and then centrifuged (410 × *g* for 1 min). An aliquot was taken from the organic phase (0.2 mL) and the aqueous phase (0.2 mL), and the ^11^C radioactivity was measured in an auto-gamma counter (Hidex AMG; Hidex, Turku, Finland). The octanol–water partition coefficient was calculated as the radioactivity ratio between the octanol and aqueous phase, and its mean logarithm (Log *P*_7.4_) was determined to express the lipophilicity.

### Tissue distribution in mice

[^11^C]**4** (9.1 MBq/51 pmol) was injected intravenously into mice (males, 9 weeks old). Mice were killed by cervical dislocation at 1, 5, 15, 30, and 60 min after injection (*n* = 4 per time point). Blood was collected following heart puncture, and the tissues were harvested. The samples were measured for ^11^C radioactivity with an auto-gamma counter (Hidex AMG) and weighed. The tissue uptake of ^11^C was expressed as the standardized uptake value (SUV): cpm measured per gram of tissue/cpm injected per gram body weight. Brain-to-blood concentration ratios of radioactivity were also calculated.

### Effect of AZM in mice

AZM increases CBF via its effects on peripheral vasodilation [23]. To investigate the increase in CBF on the tissue distribution of [^11^C]**4**, mice were pretreated with an intravenous injection of AZM (75 mg/kg) dissolved in 50% dimethylsulfoxide in physiological saline (15 mg/mL) 15 min prior to tracer injection. As a control, another group of mice was given the same volume of vehicle solution. [^11^C]**4** (9.1 MBq/13 pmol) was injected intravenously into mice, which were then killed by cervical dislocation 2 min after injection (*n* = 5). Tissue samples were handled as described above.

### Metabolite analysis in mice

[^11^C]**4** (150 MBq/1.0 nmol) was injected intravenously into mice (*n* = 3), and animals were killed by cervical dislocation 5 or 15 min later. Blood was removed following heart puncture using a heparinized syringe, and the brain was removed. The blood was centrifuged at 7000 × *g* for 1 min at 4 °C to obtain plasma. The plasma (0.2 mL) was denatured with two volumes of MeCN in an ice-water bath. The supernatant was centrifuged in the same conditions and divided into soluble and precipitate fractions. The precipitate was resuspended in two volumes of MeCN followed by centrifugation. This procedure was repeated twice. The cerebral cortex (ca. 100 mg) was homogenized in 0.5 mL H_2_O, and the homogenate was treated as described for plasma. The radioactivity in the three soluble fractions and precipitates was measured with an auto-gamma counter (Hidex AMG). The recovery yields in the soluble fraction of plasma and brain were 96.0 ± 0.8% and 98.4 ± 6.3% for 5 min after injection, and 95.3 ± 0.5% and 98.3 ± 0.2% for 15 min after injection, respectively (*n* = 3). The soluble fractions were combined, and after centrifugation of the samples as described above, a portion of the supernatant was applied to a silica gel plate (Sil60 F254, Merck Millipore) with reference standard **4**. The resultant metabolite was then separated from [^11^C]**4** with TLC using a solvent system consisting of CHCl_3_/MeOH/AcOH (84/15/1, v/v/v). TLC plates were exposed to a storage phosphor screen (GE Healthcare Life Sciences, Uppsala, Sweden) and analyzed with a phosphor imager system (Storm 820; GE Healthcare Life Sciences). Samples were compared with reference standard **4** (Rf = 0.44).

### PET study in conscious monkeys

Successive PET measurements of regional CBF with [^15^O]H_2_O and kinetics analysis of [^11^C]**4** were performed in three monkeys. To change the regional CBF, one monkey each was intravenously administered a different dose (0, 10, and 20 mg/kg) of AZM sodium (DIAMOX® Inj. 500 mg; Sanwa Kagaku Kenkyusho, Nagoya, Japan) 15 min before the [^15^O]H_2_O PET scan. We used 20 mg/kg as the highest dose for primates because this dose significantly increased the CBF in anesthetized baboons [24] without any side effects [25].

Radionuclides were produced using a HM-18 cyclotron (Sumitomo Heavy Industries) at the Central Research Laboratory of Hamamatsu Photonics. During the synthesis of [^11^C]**4** as described above, [^15^O]O_2_ production by deuteron irradiation was started, and [^15^O]H_2_O was prepared using the automated synthesis system (Sumitomo Heavy Industries).

Magnetic resonance (MR) images of the monkeys were obtained prior to the PET study with a Signa Excite HDxt 3.0T (GE Healthcare, Waukesha, WI) under anesthesia with pentobarbital. At least 1 month before the PET study, an acrylic plate that was used to fix the monkey to the monkey chair was attached to the head under pentobarbital anesthesia as described previously [26].

PET data were collected on a high-resolution animal PET scanner (SHR-38000; Hamamatsu Photonics). After an overnight fast, a venous cannula for ligand injection and an arterial cannula for blood sampling were inserted into the saphenous vein and posterior tibial artery, respectively, under temporal anesthesia using 2.5% sevoflurane in O_2_ gas. After the animal had completely recovered from the anesthesia, its head was rigidly fixed to the upper frame of a monkey chair using an acrylic head restraint. The animal, sitting in the restraining chair, was placed in the gantry at a fixed position, with stereotactic coordinates aligned parallel to the orbital plane. Following a 30-min transmission scan using a ^68^Ge–^68^ Ga rotation rod source, a bolus of [^15^O]H_2_O (1148 ± 53 MBq) was injected intravenously into the monkey, and a 2-min two-dimensional (2D) mode PET scan consisting of 12 time frames at 10-s intervals was performed. During that period, 24 arterial blood samples (0.2 mL) at 4-s intervals until 88 s and then at 120 s were withdrawn from a catheter placed in the posterior tibial artery to measure arterial radioactivity using an automated gamma counter (1480 Wizard; PerkinElmer, Waltham, MA). After an interval of 8.0–12.7 min (10.8 ± 2.0 min) from the end of the [^15^O]H_2_O PET scan, a bolus of [^11^C]**4** was administered intravenously, and dynamic PET scanning was performed in the 2D mode. The injected dose of [^11^C]**4** was 1132 ± 17 MBq (72.2 ± 19.5 nmol), the molar activity was 16.6 ± 5.0 GBq/µmol, and the radiochemical purity was > 99%.

PET images were acquired over 60 min (10 s × 6 frames, 30 s × 6 frames, 1 min × 11 frames, and 3 min × 15 frames). For quantitative analysis of [^11^C]**4**, 17 arterial blood samples (0.5 mL) were obtained every 8 s until 64 s after [^11^C]**4** injection and then at 90 s, 150 s, and 4, 6, 10, 20, 30, 45, and 60 min. Blood samples were centrifuged to separate plasma and weighed, and the radioactivity was measured using an automated gamma counter. For metabolite analysis, EtOH was added to plasma samples (EtOH/plasma = 1/1 v/v) obtained at 16, 40, and 64 s, and 6, 10, 30, 45, and 60 min after injection, followed by centrifugation. The supernatants were subjected to TLC using silica gel plates with a mobile phase of CHCl_3_/MeOH/AcOH (84/15/1, v/v/v). At each sampling time point for analysis, the ratio of radioactivity in the unmetabolized fraction to that in the total plasma (metabolized plus unmetabolized) was determined using a phosphoimaging plate (FLA-7000; Fuji Film, Tokyo, Japan). Plasma time–activity curves (pTACs) corrected for metabolites were calculated using the data obtained by correction of the ratio of the unmetabolized fraction to total radioactivity.

### PET data analysis

PET images were reconstructed using the filtered back-projection method with a Hanning filter of 4.0 mm in SHR-38000 reconstruction software (Hamamatsu Photonics), and attenuation was corrected using the transmission scan data. PET image data were analyzed using PMOD (Version 3.408; PMOD Technologies, Zurich, Switzerland). Two-dimensional regions of interest (2D-ROIs) were manually placed on the PET images with reference to the co-registered MR images. Three-dimensional regions of interest (3D-ROIs) were generated by combining 2D-ROIs. 3D-ROIs were then projected onto all frames of all dynamic PET scans. Tissue time–activity curves (tTACs) of each 3D-ROI were obtained by calculating the decay-corrected radioactivity for each frame and expressed as Bq/mL or the SUV.

PET images from 0 to 120 s after injection of [^15^O]H_2_O and the arterial input function were used to calculate regional CBF according to the method developed by Meyer [27]. Parametric images of regional CBF were generated in accordance with an autoradiographic method [28, 29].

Using the tTACs and pTACs, we evaluated the rate of [^11^C]**4** transfer from the arterial plasma to tissues (*K*_1_) and the total volume of distribution (*V*_T_) for [^11^C]**4** using the one- and two-tissue compartment models. The goodness of fit from the two-model analysis was evaluated using Akaike’s information criterion (AIC). Because short PET scans are desirable in practice, the pharmacokinetics were also explored at different acquisition times (10, 20, 30, 45, and 60 min). Parametric images of *K*_1_ and *V*_T_ were generated in accordance with the method developed by Zhou et al. [30].

### Statistical analysis

The standard deviation (SD) of the mean was used to determine the spread of data around the mean. The unpaired *t* test corrected for multiple comparison using the Holm–Sidak method was used for comparison of treatment groups with controls. The multiple *t* test with the Holm–Sidak method was used for comparison of brain and blood radioactivity for independent time points. The associations between the regional CBF and *K*_1_ obtained by [^15^O]H_2_O and [^11^C]**4** were calculated as Pearson correlation coefficients. In all analyses, *P* < 0.05 was considered to indicate statistical significance.

## Results

### Radiochemistry

The total synthesis time was within 35 min from the end of bombardment. The decay-corrected radiochemical yield of [^11^C]**4** based on [^11^C]MeI was 11.7 ± 2.7% (range, 8.3–16.7%) (*n* = 10). This lower radiochemical yield of [^11^C]**4** was caused by partial collection of the [^11^C]**4** fraction during the preparative HPLC. When collecting the entire fraction of [^11^C]**4,** the yield was 35.0 ± 12.5% (range, 27.1–49.5%) (*n* = 3). The radiochemical purity of [^11^C]**4** was 99.8 ± 0.1% (range, 99.4–99.9%) (*n* = 10). The molar activity of [^11^C]**4** was 135 ± 39.7 GBq/µmol (range, 85.3–218.1 GBq/µmol) (*n* = 10) at 30 min after the end of irradiation. Contamination with Pd in the final product was below the limit of detection (< 100 ng/mL). A very small amount of Cu was detected in two samples (120 and 260 ng/mL). This amount of Cu in the final product was far lower than what is in Cu gluconate supplementation and is not an effective dose of Cu gluconate supplementation [31].

### Octanol–water partition coefficient

The measured Log *P*_7.4_ value of [^11^C]**4** was 0.60 ± 0.02 (range, 0.56–0.64) (*n* = 6).

### Tissue distribution in mice

The tissue distributions of radioactivity after injection of [^11^C]**4** are summarized in Table 1. The blood radioactivity level rapidly decreased after injection. The lung showed the highest initial uptake followed by the kidney, heart, and pancreas. The level of radioactivity in the brain increased for the first 5 min after injection, moderately decreased until 15 min after injection, and then promptly decreased. [^11^C]**4** showed significantly higher radioactivity in the brain than in blood at all time points after injection (*P* < 0.0005, multiple *t* test with the Holm–Sidak method).

**Table 1:** Tissue distribution of [^11^C]**4** in male ddY mice

Tissue	Time after injection (min)				
	1	5	15	30	60
Blood	0.27 ± 0.04	0.26 ± 0.03	0.32 ± 0.05	0.23 ± 0.03	0.13 ± 0.02
Heart	1.91 ± 0.14	0.91 ± 0.07	0.54 ± 0.02	0.36 ± 0.04	0.18 ± 0.03
Lung	12.61 ± 1.18	6.10 ± 0.88	2.85 ± 0.39	1.24 ± 0.20	0.67 ± 0.05
Liver	0.43 ± 0.12	1.39 ± 0.25	1.77 ± 0.24	1.24 ± 0.05	0.81 ± 0.03
Pancreas	1.32 ± 0.23	2.49 ± 0.19	2.39 ± 0.33	1.30 ± 0.15	0.62 ± 0.30
Spleen	0.92 ± 0.12	1.52 ± 0.32	1.32 ± 0.11	0.66 ± 0.08	0.33 ± 0.07
Small intestine	1.29 ± 0.14	1.51 ± 0.15	1.32 ± 0.25	0.68 ± 0.06	0.35 ± 0.08
Kidney	4.28 ± 1.05	4.70 ± 0.38	2.69 ± 0.61	1.64 ± 0.20	0.98 ± 0.09
Muscle	0.63 ± 0.11	0.69 ± 0.08	0.44 ± 0.05	0.27 ± 0.04	0.21 ± 0.08
Brain	1.13 ± 0.23	1.69 ± 0.12	1.44 ± 0.11	0.75 ± 0.12	0.34 ± 0.04
Brain/blood	4.16 ± 0.60	6.54 ± 0.50	4.51 ± 0.52	3.29 ± 0.37	2.61 ± 0.52

The radioactivity levels are expressed as SUVs

Data represent the mean ± SD (*n* = 4)

### Effects of AZM

Table 2 summarizes the effects of AZM on the tissue distribution at 2 min after injection of [^11^C]**4**. Pretreatment with AZM increased the brain radioactivity to 136.8% (*P* < 0.05) and decreased lung radioactivity to 51.0% (*P* < 0.05) compared to vehicle-pretreated mice. Blood levels of radioactivity were not changed in the AZM-pretreated group or vehicle-pretreated group. As a result, the brain-to-blood ratio was significantly increased to 144.9% (*P* < 0.05) in the AZM-pretreated group.

**Table 2:** Effect of acetazolamide on the distribution of [^11^C]**4** in male ddY mice 2 min after injection

Tissue	Vehicle	Acetazolamide	Increase (%)	*P*
Blood	0.35 ± 0.06	0.33 ± 0.03	94.3	N.S.
Heart	1.62 ± 0.37	1.51 ± 0.20	93.2	N.S.
Lung	8.99 ± 1.96	4.58 ± 1.44	51.0	< 0.05
Liver	0.78 ± 0.10	1.05 ± 0.16	134.6	N.S.
Kidney	6.46 ± 0.99	5.85 ± 1.21	90.6	N.S.
Brain	1.90 ± 0.36	2.60 ± 0.13	136.8	< 0.05
Brain/blood	5.53 ± 1.12	8.01 ± 0.79	144.9	< 0.05

The radioactivity levels are expressed as SUVs

Data represent the mean ± SD (*n* = 5)

Unpaired *t* test corrected for multiple comparison using the Holm–Sidak method

*N.S.* not significant

### Metabolite analysis

Table 3 summarizes the percentages of radiolabeled metabolites in the plasma and brain at 5 and 15 min after intravenous injection of [^11^C]**4**. The hydrophilic metabolite (HM) with an Rf = 0.25 was mainly detected (Additional file 1: Figure S1a and b) in plasma. A minor lipophilic component of Rf = 0.63 was only observed in plasma. [^11^C]**4** was rapidly metabolized to the HM in plasma, and only 14.8 ± 2.2% (*n* = 3) existed in the unchanged form at 15 min after injection. In contrast, over 99% of the radioactivity in the brain was intact at 5 and 15 min after injection (Additional file 1: Figure S1c and d).

**Table 3:** Metabolite analysis of [^11^C]**4** in male ddY mice

Components	Rf value	Plasma metabolites (%)		Brain metabolites (%)	
		5 min	15 min	5 min	15 min
HM1	0.09	7.87 ± 2.17	26.65 ± 3.40	0.03 ± 0.02	0.03 ± 0.02
HM2	0.25	53.25 ± 6.59	59.06 ± 6.13	0.01^a^	0.13 ± 0.02
HM3	0.36	0.54 ± 0.26	0.04^a^	0.09^a^	0.02^a^
Unchanged	0.44	38.29 ± 6.52	14.76 ± 2.23	99.87 ± 0.03	99.84 ± 0.04
LM	0.63	0.05 ± 0.03	0.01 ± 0.01	N.D.	N.D.

Data represent the mean ± SD (*n* = 3)

*N.D.* not detected, *HM* hydrophilic metabolite, *LM* lipophilic metabolite

^a^Detected in two samples

### Monkey PET study

**Fig. 3:**
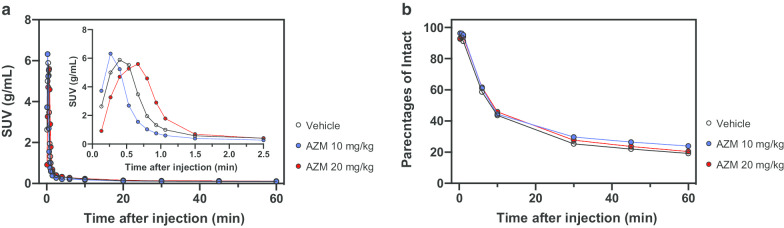
Decay-corrected TACs of metabolite-corrected plasma (**a**) and percentage of intact [^11^C]**4** (**b**) after intravenous injection of [^11^C]**4** into monkeys. Values for 2.5 min were extracted from **a** and inserted

**Fig. 4:**
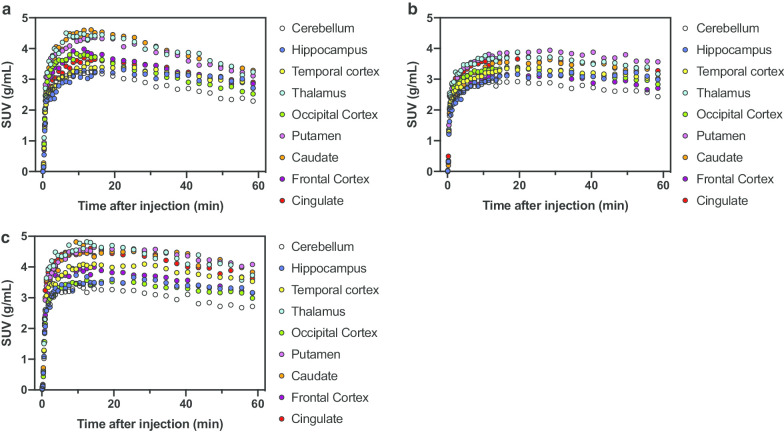
Decay-corrected TACs of nine brain regions after intravenous injection of [^11^C]**4** into monkeys pretreated with vehicle (**a**), 10 mg/kg AZM (**b**), and 20 mg/kg AZM (**c**). Nine brain regions were selected to generate tTACs: cerebellum, merged bilateral hippocampus, merged bilateral temporal cortex, thalamus, merged bilateral occipital cortex, merged bilateral putamen, merged bilateral caudate, merged bilateral frontal cortex, and cingulate

Plasma radioactivity rapidly decreased after a bolus injection of [^11^C]**4** (Fig. 3a). At 30 min after injection, the parent fraction of plasma radioactivity was 27.6 ± 2.2% (*n* = 3) (Fig. 3b). [^11^C]**4** metabolism in monkeys was slower than that in mice. The tTACs in the nine 3D-ROIs of [^11^C]**4** are presented in Fig. 4. In the vehicle-treated monkey, radioactivity in the nine 3D-ROIs peaked at 6.5–16.5 min and then slowly decreased (Fig. 4a). In the monkey treated with 10 mg/kg AZM, radioactivity peaks were delayed and retained thereafter (Fig. 4b). However, tTACs of the monkey treated with 20 mg/kg AZM were similar to those of the vehicle-treated monkey (Fig. 4c). The increase in the tTACs at the initial phase reflected the initial input function (Additional file 1: Figure S2a). The differences in the tTACs after the initial phase may reflect the input released from the lung. As indicated in the mouse biodistribution study, [^11^C]**4** was first trapped in the lung and then gradually released into the blood (Table 1), similar to the parent compound, [^123^I]IMP. The clearance rate of metabolite-corrected pTACs in the later phase was similar in both the vehicle- and 20 mg/kg AZM-treated monkeys, but faster than that in the 10 mg/kg AZM-treated monkey (Additional file 1: Figure S2b). This slower clearance of the input function in the later phase is likely the cause of the slower brain kinetics in the 10 mg/kg AZM-treated monkey.

**Fig. 5:**
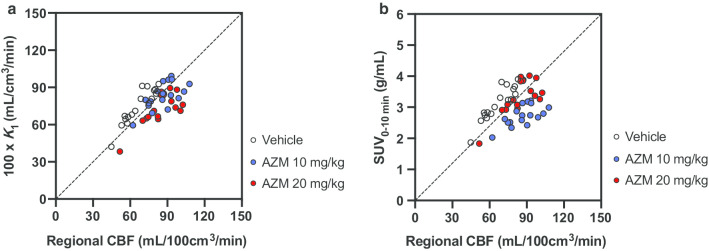
Comparison of regional CBF values obtained by [^15^O]H_2_O and *K*_1_ (**a**) and early phase SUV (**b**) of [^11^C]**4**. *K*_1_ values of (**a**) were multiplied by 100 to align with the units of CBF. Seventeen brain regions were selected for comparison: cerebellum, right and left hippocampus, right and left temporal cortex, hypothalamus, thalamus, right and left occipital cortex, right and left putamen, right and left caudate, right and left frontal cortex, cingulate, and white matter

**Fig. 6:**
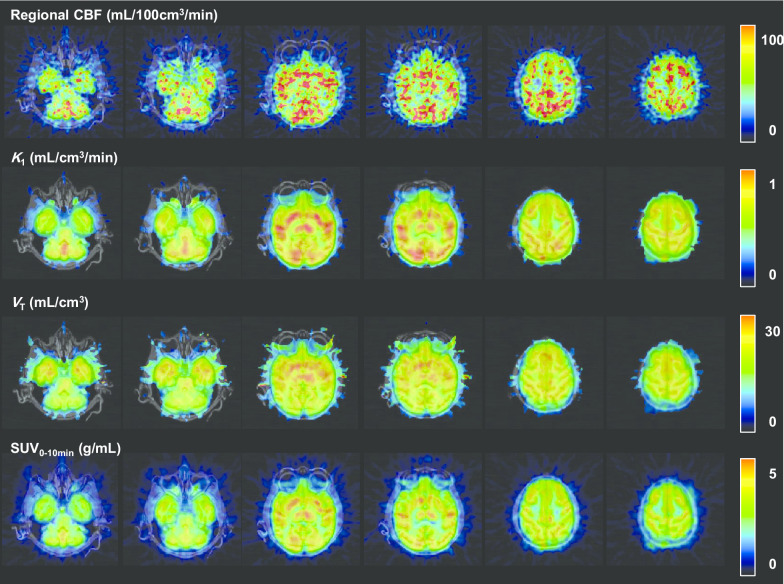
MR-fused parametric images of the vehicle-pretreated monkey. Regional CBF images of [^15^O]H_2_O were calculated using autoradiography [28, 29]. Parametric images of [^11^C]**4** were calculated using a 45-min scan duration and one-tissue compartment model [30]. Static images acquired from 0 to 10 min after injection of [^11^C]**4** were expressed as the SUV

The preliminary kinetics analysis of the comparison of AIC (paired *t* test, *P* < 0.05) in all regions investigated showed that the one-tissue compartment model provided significantly better AIC scores than the two-tissue compartment model (*n* = 3). Additional file 1: Table S1 summarizes the *K*_1_, *V*_T_, and regional CBF values of selected brain regions in the three individual monkeys. The regional *K*_1_ values of [^11^C]**4** calculated with a 45-min scan duration in the vehicle-treated monkey were well correlated with that of regional CBF (*r* = 0.9230, *P* < 0.0001) (Fig. 5a). In contrast, no correlation with regional CBF was observed for *V*_T_ at any duration of acquisition times. Kinetic analysis did not identify significant differences among the *K*_1_ values calculated with different acquisition times, but the 45-min scan duration was slightly better correlated with regional CBF than the others (10 min: *r* = 0.9015, *P* < 0.0001 vs. 45 min: *r* = 0.9230, *P* < 0.0001). Furthermore, the SUV from the early phase scan (0–10 min) showed good correlation with regional CBF (*r* = 0.9042, *P* < 0.0001) (Fig. 5b). In contrast, the SUV from the later phase scan was not good for evaluating regional CBF (0–10 min: *r* = 0.9042, *P* < 0.0001 vs. 40–60 min: *r* = 0.5673, *P* = 0.0184). These data suggest that [^11^C]**4** probably detects changes in regional CBF in the low-to-normal range of flows. AZM treatment increased the mean total ROIs of CBF to 133%, although dose dependency was not observed. In contrast, 10 mg/kg AZM treatment slightly increased the mean total ROIs of *K*_1_ to 114%. However, 20 mg/kg AZM treatment did not show an increase in *K*_1_ (101%). The correlation between *K*_1_ and regional CBF was decreased at a higher flow range with AZM loading (10 mg/kg: *r* = 0.6581, *P* = 0.0041; 20 mg/kg: *r* = 0.7510, *P* = 0.0005) due to underestimation of regional CBF at higher flows. Figure 6 shows the representative parametric PET/MR-fused images of a vehicle-treated monkey brain acquired after injection of [^11^C]**4**. Parametric images of *K*_1_ and SUV_0–10 min_ closely resembled those of regional CBF images: Higher radioactivity accumulation was observed in frontal and occipital cortexes, thalamus, striatum, and cingulate. In contrast, the *V*_T_ image was rather homogeneous and showed higher radioactivity accumulation in the temporal cortex, thalamus, and striatum.

## Discussion

The palladium-mediated reaction of stannanes with [^11^C]MeI employing Stille cross-coupling reactions was successfully applied to synthesize [^11^C]**4**. In this study, we investigated fundamental biological properties of [^11^C]**4** as a CBF tracer in the brains of mice and conscious monkeys. Although [^11^C]**4** had lower measured Log *P*_7.4_ values and rapid degradation in the periphery, [^11^C]**4** was rapidly incorporated into the brain and moderately decreased until 15 min after tracer injection in mice. In the monkey brain, [^11^C]**4** was widely distributed in gray matter regions. The brain kinetics of [^11^C]**4** in the monkey brain was slower than that in mouse brain due to the slower peripheral metabolism of [^11^C]**4** in monkeys. The local distribution pattern of [^11^C]**4** in the monkey brain closely resembled that of [^15^O]H_2_O. Furthermore, AZM treatment significantly increased the brain radioactivity, and the brain-to-blood ratio in mice indicated that [^11^C]**4** may reflect changes in CBF. However, this change was low in the monkey brain because the dose of AZM administered to one monkey (20 mg/kg) was lower than in mice (75 mg/kg). Taken together, these fundamental properties of [^11^C]**4** may suggest the feasibility of [^11^C]**4** as a novel CBF PET tracer.

Development of alternative CBF PET tracers instead of [^15^O]H_2_O, such as freely permeable alcohol [32], volatile fluoroalkanes [33–35], and antipyrines [36] (detailed summary is described in the textbook [37]), involves several challenges. However, these diffusible tracers have not been widely used since retention-type SPECT tracers have become available. Retention-type PET tracers labeled with unconventional generator-produced ^62^Cu radionuclides named [^62^Cu]Cu-pyruvaldehyde-bis(*N*^4^-methylthiosemicarbazone) ([^62^Cu]Cu-PTSM) were developed by Green and colleagues [38, 39]. A direct comparison of [^62^Cu]Cu-PTSM and [^15^O]H_2_O showed that the relative distribution of [^62^Cu]Cu-PTSM correlates well with regional CBF obtained with [^15^O]H_2_O [40]. One serious limitation to the use of [^62^Cu]Cu-PTSM is the lack of a commercial supply of ^62^Zn/^62^Cu generators. In the meantime, hundreds of cyclotrons have been installed worldwide and are used to produce conventional PET radionuclides for routine nuclear medicine practice. Based on these facts, novel retention-type CBF PET tracers labeled with conventional PET radionuclides are expected to play important roles in clinical practice. Kizuka and co-workers have prepared *N*-[^11^C-methyl]chlorphentermine ([^11^C]NMCP) and *N*,*N*-[^11^C-dimethyl]chlorphentermine ([^11^C]NDMCP) as potential CBF PET tracers [41]. They concluded that [^11^C]NMCP is a potential CBF PET imaging agent because of its longer retention in the brain. Although preliminary biodistribution data of [^11^C]NMCP showed comparable uptake and retention to that of [^123^I]IMP in the rat brain, further detailed evaluations such as metabolism, brain kinetics in larger animals, and linearity of the CBF range have not been reported. The retention mechanisms of [^11^C]NMCP are related to the interaction of chlorphentermine structures with tissue phospholipids [42], which cause unfavorable nonspecific binding of this tracer to the white matter. This will reduce the gray-to-white matter contrast. Furthermore, the calculated SUV of [^11^C]NMCP (3.0) was lower than that of [^11^C]**4** (4.2) in rats (Additional file 1: Figure S3).

From this preliminary study, use of [^11^C]**4** as a CBF tracer has some limitations. One is the lower lipophilicity of [^11^C]**4** (Log *P*_7.4_ = 0.6), which may limit free diffusion of the tracer across the blood–brain barrier. This may be the cause of the initial low uptake of [^11^C]**4** because this suboptimal lipophilicity lowers the first pass extraction rate. In contrast, the log *P* value of the standard CBF SPECT tracer [^123^I]IMP is 1.6 [43]. We used the final product of [^11^C]**4**, which contained 0.125% solubilizing agent (polysorbate 80), for log *P*_7.4_ analysis. Although the calculated amount of the reagent was very low (0.0003% in the assay mixture), the presence of this small amount of solubilizer may increase the solubility of [^11^C]**4** in the aqueous phase. A second limitation is the rapid metabolism of [^11^C]**4** in peripheral organs, which will reduce the input. Third, we observed gradual washout of [^11^C]**4** from the brain. This problem is crucial in SPECT, which requires 20–30 min of data acquisition time with the rotating gamma camera. However, this limitation may not be a problem for ring-type PET scanners. These second and third limitations are closely related because washout kinetics from the brain could be reflected by the metabolic rate of [^11^C]**4** in the periphery. Indeed, washout kinetics in conscious monkeys was not rapid, although rodents showed rapid washout kinetics from the brain (Additional file 1: Figure S3b). The most critical limitation is the loss of a good correlation between regional CBF and *K*_1_ values of [^11^C]**4** after AZM loading in monkeys. We speculate that this is due to underestimation of regional CBF at a higher flow rate. However, technically, individual differences in brain kinetics in this small-sized monkey PET study may have obscured clear understanding of these results. Furthermore, monkeys may feel stress and change their respiration rate during the PET scanning, which will influence the regional CBF. Practically, achieving perfect control of the resting state in conscious monkeys is difficult. Monitoring the partial pressure of CO_2_ and O_2_, pH, heart rate, and arterial blood pressure is mandatory not only to verify the effect of AZM treatment but also to perform reliable measurement of regional CBF. Therefore, further validation studies with a verified PET scanning protocol and a larger number of individuals are needed.

Although [^11^C]**4** underestimated regional CBF at a higher flow rate, it can be used to detect low flow regions of impaired brain function. To confirm this hypothesis, further validation studies of [^11^C]**4** in aged monkeys [44] and/or cerebral ischemia models [45] will be useful. Furthermore, the SUV of the early phase scan (0–10 min) showed a good correlation with regional CBF. Because short PET scans are desirable in practice, [^11^C]**4** may be useful instead of [^18^F]FDG for diagnosis of dementia in patients with DM.

## Conclusion

[^11^C]**4** was prepared by palladium-mediated Stille coupling reactions. [^11^C]**4** has potential as a novel CBF PET tracer. To confirm the clinical availability of [^11^C]**4**, further validation studies in aged monkeys and/or cerebral ischemia models are needed.

## Supplementary information

**Additional file 1:** **Figure S1.** Representative radio-thin-layer chromatogram of plasma (**a** and **b**) and brain (**c** and **d**) samples at 5 (**a** and **c**) and 15 (**b** and **d**) min after injection of [^11^C]**4** into ddY mice. **Figure S2.** Decay-corrected TACs of the whole brain after intravenous injection of [^11^C]**4** into the monkeys. Values from 0–2.3 min are indicated (**a**). Decay-corrected TACs of metabolite-corrected plasma after intravenous injection of [^11^C]**4** into monkeys. Values after 2.5 min were extracted from Figure 3a. (**b**). **Figure S3.** Representative static images acquired from 1.25 to 6.5 min after injection of [^11^C]**4** in a Wistar rat expressed as standardized uptake values (range, 0.5–5 g/mL) (**a**). PET images were co-registered on MR images. Time-activity curves of the whole brain region of a Wistar rat after injection of [^11^C]**4** (*n* = 3) (**b**). Brain radioactivity increased for the first 1.75 min after [^11^C]**4** injection, was maintained until 6.5 min, and then promptly decreased thereafter. **Table S1.** *K*_1_,*V*_T_, and regional CBF values of selected brain regions in rhesus monkeys.

## Data Availability

The datasets used and/or analyzed during the current study are available from the corresponding author on reasonable request.

## Abbreviations

AcOH: Acetic acid
AcONH_4_: Ammonium acetate
AD: Alzheimer’s disease
AIC: Akaike’s information criterion
AZM: Acetazolamide
CBF: Cerebral blood flow
Cu-PTSM: Cu-pyruvaldehyde-bis(*N*^4^-methylthiosemicarbazone)
DM: Diabetes mellitus
DMF: *N*,*N*-Dimethylformamide
EtOAc: Ethyl acetate
EtOH: Ethanol
[^18^F]FDG: 2-Deoxy-2-[^18^F]fluoro-d-glucose
HM: Hydrophilic metabolite
HPLC: High-performance liquid chromatography
HRMS: High-resolution mass spectrometry
i.d.: Inner diameter
IMP: *N*-Isopropyl-*p*-iodoamphetamine
*K*_1_: Arterial plasma-to-tissue rate constant
LM: Lipophilic metabolite
MeCN: Acetonitrile
MeI: Methyl iodide
MeOH: Methanol
MR: Magnetic resonance
N.D.: Not detected
NIH: National Institutes of Health
NMR: Nuclear magnetic resonance
N.S.: Not significant
PET: Positron emission tomography
PG: Plasma glucose
pTACs: Plasma time-activity curves
Rf: Retention fraction
ROIs: Regions of interest
r.t.: Retention time
SPECT: Single photon emission computed tomography
SD: Standard deviation
SUV: Standardized uptake value
TLC: Thin-layer chromatography
2D: Two-dimensional
3D: Three-dimensional
tTACs: Tissue time-activity curves
UV: Ultraviolet
*V*_T_: Total volume of distribution
