# Supplementary material for: Synthesis and evaluation of N-isopropyl-p-[11C]methylamphetamine as a novel cerebral blood flow tracer for positron emission tomography
Source: EJNMMI Res. 2020 Oct 1;10:115. doi: 10.1186/s13550-020-00702-5 (PMC7527401; doi:10.1186/s13550-020-00702-5)
Supplement: Supplementary file 1 — Additional file 1: Figure S1. Representative radio-thin-layer chromatogram of plasma (a and b) and brain (c and d) samples at 5 (a and c) and 15 (b and d) min after injection of [11C]4 into ddY mice. Figure S2. Decay-corrected TACs of the whole brain after intravenous injection of [11C]4 into the monkeys. Values from 0–2.3 min are indicated (a). Decay-corrected TACs of metabolite-corrected plasma after intravenous injection of [11C]4 into monkeys. Values after 2.5 min were extracted from Figure 3a. (b). Figure S3. Representative static images acquired from 1.25 to 6.5 min after injection of [11C]4 in a Wistar rat expressed as standardized uptake values (range, 0.5–5 g/mL) (a). PET images were co-registered on MR images. Time-activity curves of the whole brain region of a Wistar rat after injection of [11C]4 (n = 3) (b). Brain radioactivity increased for the first 1.75 min after [11C]4 injection, was maintained until 6.5 min, and then promptly decreased thereafter. Table S1. K1,VT, and regional CBF values of selected brain regions in rhesus monkeys. [file 13550_2020_702_MOESM1_ESM.docx]

**Supplementary Information**

**Figure S1.** Representative radio-thin-layer chromatogram of plasma (**a** and **b**) and brain (**c** and **d**) samples at 5 (**a** and **c**) and 15 (**b** and **d**) min after injection of [^11^C]**4** into ddY mice.

**Figure S2.** Decay-corrected TACs of the whole brain after intravenous injection of [^11^C]**4** into the monkeys. Values from 0–2.3 min are indicated (**a**). Decay-corrected TACs of metabolite-corrected plasma after intravenous injection of [^11^C]**4** into monkeys. Values after 2.5 min were extracted from Figure 3a. (**b**).

*Small animal PET imaging:* Wistar rats were purchased from Japan SLC (Hamamatsu, Japan). The animals were allowed to acclimate to the laboratory environment for at least 1 week prior to use. The Animal Care and Use Committee of the Tokyo Metropolitan Institute of Gerontology approved the animal studies (Approval No. 18015). Rats (males, 8 weeks old) were not fasted prior to the PET scan. Rats (*n* = 3) were scanned dynamically with the brain in the field of view using a semiconductor small animal PET scanner (MIP-100; Sumitomo Heavy Industries, Tokyo, Japan) [1]. Rats were anesthetized with a mixture of isoflurane/air (inhalation anesthesia, 5% ratio during induction, later reduced to <2%). Rats were confirmed to be under anesthesia before tracer injection. [^11^C]**4** (183 ± 28 MBq/2.1 ± 0.8 nmol) injection was performed through a catheter placed in the tail vein after careful positioning of the animals in the PET scanner. A list-mode protocol was used for 60 min. Scanning was started at the time of injection of radioactivity into the rat. The rats were sacrificed by cervical dislocation after the PET scan.

List-mode data were reframed into a dynamic sequence of frames of 8 × 30 s, 3 × 60 s, 2 × 120 s, 2 × 180 s, 3 × 300 s, 2 × 540 s, and 1 × 600 s. The data were reconstructed per time frame using an iterative reconstruction algorithm (three-dimensional ordered-subset expectation maximization, provided by Sumitomo Heavy Industries; one iteration, 32 subsets). The final datasets consisted of 31 slices, with a slice thickness of 0.85 mm and an in-plane image matrix of 256 × 256 pixels of size 0.3 × 0.3 mm. Datasets were fully corrected for random coincidences and scatter. Images were smoothed with a Gaussian filter (1.5 mm in both transverse and axial directions).

Anatomical images of the rat brain were generated with an ICON 1 T benchtop magnetic resonance imaging (MRI) system (Bruker BioSpin, Ettlingen, Germany). Coronal T2-weighted MRI was performed with a fast spin-echo sequence with the following image parameters: repetition time = 3432.2 ms, effective echo time = 60 ms, field of view = 35 × 35 mm, and slice thickness = 1.25 mm.

*Small animal PET data analysis:* PET image data were analyzed using standard software (PMOD, version 3.408; PMOD Technologies, Zurich, Switzerland). PET images of the summed scan were manually registered on the MR images of the rat brain. Two-dimensional regions of interest (2D-ROIs) were carefully drawn in each slice to cover entire brain areas with reference to the co-registered MR images. Three-dimensional regions of interest (3D-ROIs) were generated by combining 2D-ROIs. 3D-ROIs were then projected onto all frames of all dynamic PET scans, resulting in time–activity curves for each scan and rat. PET standardized uptake values were calculated using measured body weights and injected doses and assuming a specific gravity of 1 g/mL for tissue.

**Figure S3.** Representative static images acquired from 1.25 to 6.5 min after injection of [^11^C]**4** in a Wistar rat expressed as standardized uptake values (range, 0.5−5 g/mL) (**a**). PET images were co-registered on MR images. Time-activity curves of the whole brain region of a Wistar rat after injection of [^11^C]**4** (*n* = 3) (**b**). Brain radioactivity increased for the first 1.75 min after [^11^C]**4** injection, was maintained until 6.5 min, and then promptly decreased thereafter.

**Reference**

1. Ishii K, Kikuchi Y, Matsuyama S, Kanai Y, Kotani K, Ito T, Yamazaki H, Funaki Y, Iwata R, Itoh M, Yanai K, Hatazawa J, Itoh N, Tanizaki D, Amano D, Yamada M, Yamaguchi T. First achievement of less than 1 mm FWHM resolution in practical semiconductor animal PET scanner. Nucl Instrum Meth A. 2007;576:435–40.

**Table S1.** *K*_1_, *V*_T_, and regional CBF values of selected brain regions in rhesus monkeys

| region | Regional CBF (mL/100 cm^3^/min) | | | *K*_1_ (mL/cm^3^/min)* | | | *V*_T_ (mL/cm^3^)* | | |
| --- | --- | --- | --- | --- | --- | --- | --- | --- | --- |
|  | Vehicle | AZM 10 mg/kg | AZM 20 mg/kg | Vehicle | AZM 10 mg/kg | AZM 20 mg/kg | Vehicle | AZM 10 mg/kg | AZM 20 mg/kg |
| Cerebellum | 61.33 | 76.60 | 73.22 | 0.68 | 0.79 | 0.65 | 18.47 | 22.19 | 18.25 |
| Hippocampus-right | 57.63 | 74.81 | 74.51 | 0.61 | 0.75 | 0.66 | 24.32 | 28.67 | 22.88 |
| Hippocampus-left | 53.09 | 90.36 | 70.01 | 0.59 | 0.72 | 0.63 | 22.72 | 27.66 | 22.01 |
| Temporal cortex-right | 58.27 | 92.84 | 102.42 | 0.66 | 0.84 | 0.76 | 24.39 | 27.40 | 25.64 |
| Temporal cortex-left | 56.15 | 86.27 | 96.51 | 0.64 | 0.85 | 0.74 | 22.98 | 27.39 | 24.07 |
| Hypothalamus | 55.54 | 77.75 | 100.61 | 0.67 | 0.69 | 0.71 | 21.45 | 24.68 | 24.12 |
| Thalamus | 69.64 | 93.17 | 92.15 | 0.91 | 0.99 | 0.89 | 27.16 | 29.58 | 26.87 |
| Occipital cortex-right | 80.85 | 103.39 | 82.60 | 0.85 | 0.87 | 0.64 | 20.00 | 25.15 | 20.74 |
| Occipital cortex-left | 77.34 | 107.77 | 82.35 | 0.81 | 0.93 | 0.67 | 20.02 | 26.64 | 21.29 |
| Putamen-right | 73.83 | 86.54 | 87.14 | 0.91 | 0.95 | 0.85 | 26.21 | 33.29 | 28.66 |
| Putamen-left | 79.31 | 90.86 | 86.94 | 0.87 | 0.96 | 0.84 | 25.62 | 33.46 | 28.01 |
| Caudate-right | 82.99 | 81.79 | 85.24 | 0.93 | 0.87 | 0.86 | 29.04 | 32.63 | 27.73 |
| Caudate-left | 80.35 | 99.12 | 85.07 | 0.89 | 0.81 | 0.84 | 26.64 | 30.94 | 27.19 |
| Frontal cortex-right | 68.62 | 72.31 | 93.15 | 0.81 | 0.80 | 0.79 | 22.15 | 26.32 | 22.07 |
| Frontal cortex-left | 74.89 | 85.92 | 79.17 | 0.77 | 0.80 | 0.71 | 23.22 | 24.67 | 21.99 |
| Cingulate | 64.03 | 93.62 | 97.70 | 0.71 | 0.96 | 0.88 | 23.85 | 29.49 | 25.34 |
| White matter | 44.79 | 62.14 | 51.75 | 0.42 | 0.59 | 0.38 | 19.48 | 23.87 | 19.03 |

*Results of a 45-min scan
